# (μ-3,5,9,11-Tetra­oxo-4,10-diaza­tetra­cyclo­[5.5.2.0^2,6^.0^8,12^]tetra­dec-13-ene-4,10-diido-κ^2^ *N*:*N*′)bis­[(2,2′-bipyridine-κ^2^ *N*,*N*′)silver(I)] dihydrate

**DOI:** 10.1107/S1600536812038640

**Published:** 2012-09-15

**Authors:** Yongmei Zhang

**Affiliations:** a School of Chemistry and Life Science, Anshan Normal University, Anshan, Liaoning 114000, People’s Republic of China

## Abstract

In the title complex, [Ag_2_(C_12_H_8_N_2_O_4_)(C_10_H_8_N_2_)_2_]·2H_2_O, the Ag^I^ ion is three-coordinated by two N atoms from a chelating 2,2′-bipyridine ligand and one N atom from an imide ligand in a Y-shaped fashion. The imide ligand and the complex lie on a twofold rotation axis. The ligand bridges two Ag^I^ ions, forming a dinuclear complex. In the crystal, O—H⋯O hydrogen bonds link the lattice water mol­ecules and the complex mol­ecules into a ribbon-like structure along [001]. π–π inter­actions are observed between the pyridine rings [centroid–centroid distance = 3.8289 (14) Å].

## Related literature

For structures and properties of mixed-ligand coordination polymers, see: Song *et al.* (2012 ▶); Wang (2010 ▶). For the use of mol­ecular building blocks associated with polydentate carb­oxy­lic acids, see: Liao *et al.* (2008 ▶); Wang *et al.* (2009 ▶).
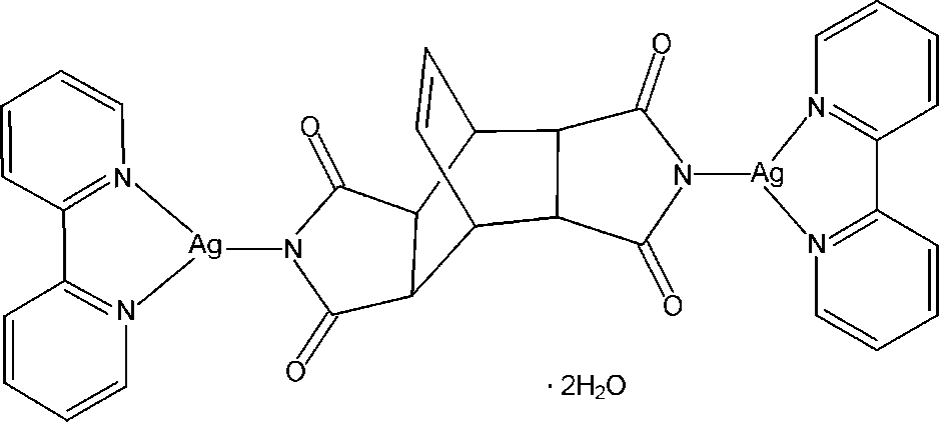


## Experimental

### 

#### Crystal data

[Ag_2_(C_12_H_8_N_2_O_4_)(C_10_H_8_N_2_)_2_]·2H_2_O
*M* *_r_* = 808.34
Monoclinic, 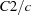

*a* = 22.2720 (12) Å
*b* = 7.1013 (4) Å
*c* = 19.6329 (11) Å
β = 108.376 (1)°
*V* = 2946.8 (3) Å^3^
*Z* = 4
Mo *K*α radiation
μ = 1.39 mm^−1^
*T* = 293 K
0.24 × 0.22 × 0.21 mm

#### Data collection

Bruker APEXII CCD diffractometer
Absorption correction: multi-scan (*SADABS*; Bruker, 2001 ▶) *T*_min_ = 0.712, *T*_max_ = 0.758
7869 measured reflections
2923 independent reflections
2588 reflections with *I* > 2σ(*I*)
*R*_int_ = 0.020

#### Refinement

*R*[*F* ^2^ > 2σ(*F* ^2^)] = 0.021
*wR*(*F* ^2^) = 0.051
*S* = 1.03
2923 reflections
214 parameters
2 restraints
H atoms treated by a mixture of independent and constrained refinement
Δρ_max_ = 0.30 e Å^−3^
Δρ_min_ = −0.32 e Å^−3^

### 

Data collection: *APEX2* (Bruker, 2007 ▶); cell refinement: *SAINT* (Bruker, 2007 ▶); data reduction: *SAINT*; program(s) used to solve structure: *SHELXTL* (Sheldrick, 2008 ▶); program(s) used to refine structure: *SHELXTL*; molecular graphics: *XP* in *SHELXTL*; software used to prepare material for publication: *SHELXTL*.

## Supplementary Material

Crystal structure: contains datablock(s) global, I. DOI: 10.1107/S1600536812038640/hy2587sup1.cif

Structure factors: contains datablock(s) I. DOI: 10.1107/S1600536812038640/hy2587Isup2.hkl

Additional supplementary materials:  crystallographic information; 3D view; checkCIF report

## Figures and Tables

**Table 1:** Hydrogen-bond geometry (Å, °)

*D*—H⋯*A*	*D*—H	H⋯*A*	*D*⋯*A*	*D*—H⋯*A*
O1*W*—H1*A*⋯O2	0.81 (3)	2.07 (2)	2.839 (2)	159 (3)
O1*W*—H1*B*⋯O2^i^	0.82 (3)	2.13 (3)	2.952 (3)	175 (2)

Symmetry code: (i) 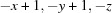.

**Fig. 1.**
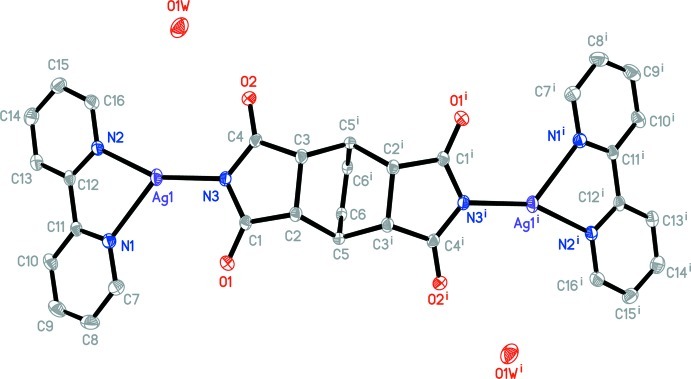
The molecular structure of the title compound. Displacement ellipsoids are drawn at the 30% probability level. [Symmetry code: (i) 1-x, y, 1/2-z.]

**Fig. 2.**
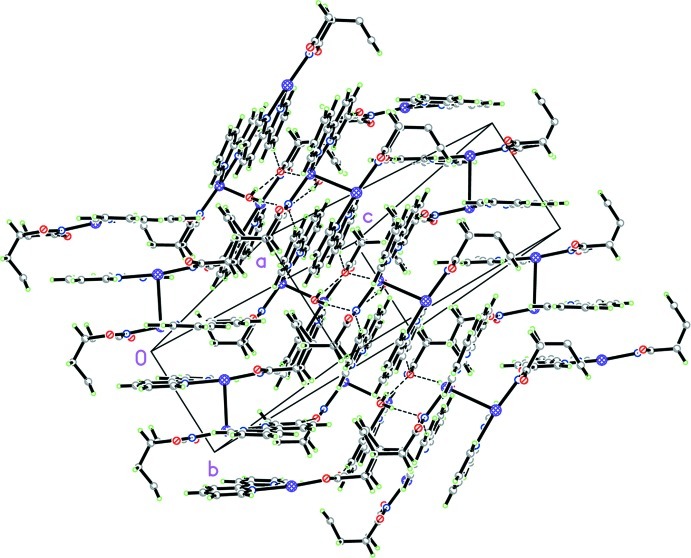
View of the crystal packing of the title compound. Hydrogen bonds are shown as dashed lines.

## supplementary crystallographic information

### Comment

The assembly of mixed-ligand coordination polymers has attracted great attention due to their intriguingly complicated architectures and potential applications in adsorption, separation and magnetism (Song *et al.*, 2012; Wang, 2010). Molecular building blocks associated with polydentate carboxylic acids are widely used in chiral catalysis, optoelectronic materials, hematopathology and medicine (Liao *et al.*, 2008; Wang *et al.*, 2009). In our laboratory, we synthesized a new silver(I) complex constructed by an amide molecule in combination with 2,2-bipyridine as ancillary ligand.

In the title complex, the amide ligand lies on a twofold rotation axis and bridges two 2,2'-bipyridine-chelated Ag^I^ atoms. The Ag^I^ atom is three-coordinated by two N atoms of a 2,2'-bipyridine ligand [Ag—N distances = 2.3510 (18) and 2.2380 (18) Å] and one N atom from an amide ligand [Ag—N distance = 2.1123 (17) Å]. In the crystal, O—H···O hydrogen bonds link the uncoordinated water molecules and the complex molecules into a ribbon-like structure. π–π interactions between the pyridine rings are observed [centroid–centroid distance = 3.8289 (14) Å].

### Experimental

A mixture of bicyclo[2,2,2]oct-7-ene-2,3,5,6-tetracarboxylic dianhydride (0.1 mmol, 0.025 g), 2,2'-bipyridine (0.2 mmol, 0.080 g), silver nitrate (0.2 mmol, 0.034 g) and H_2_O (15 ml) was stirred for ten minutes. Dilute ammonia was dropwised into the mixture until the mixture turned to transparent. Colorless block crystals of the title compound were isolated after the evaporation of ammonia.

### Refinement

H atoms on C atoms were positioned geometrically and refined as riding atoms, with C—H = 0.93 and 0.98 Å and with *U*_iso_(H) = 1.2*U*_eq_(C). H atoms bonded to O atoms were located in a difference Fourier map and refined with O—H distance restraints of 0.85 (2) Å and with *U*_iso_(H) = 1.5*U*_eq_(O).

### Crystal data

**Table:**

[Ag_2_(C_12_H_8_N_2_O_4_)(C_10_H_8_N_2_)_2_]·2H_2_O	*F*(000) = 1616
*M**_r_* = 808.34	*D*_x_ = 1.822 Mg m^−^^3^
Monoclinic, *C*2/*c*	Mo *K*α radiation, λ = 0.71073 Å
Hall symbol: -C 2yc	Cell parameters from 2928 reflections
*a* = 22.2720 (12) Å	θ = 1.0–26.1°
*b* = 7.1013 (4) Å	µ = 1.39 mm^−^^1^
*c* = 19.6329 (11) Å	*T* = 293 K
β = 108.376 (1)°	Block, colorless
*V* = 2946.8 (3) Å^3^	0.24 × 0.22 × 0.21 mm
*Z* = 4	

### Data collection

**Table:**

Bruker APEXII CCD diffractometer	2923 independent reflections
Radiation source: fine-focus sealed tube	2588 reflections with *I* > 2σ(*I*)
Graphite monochromator	*R*_int_ = 0.020
φ and ω scans	θ_max_ = 26.1°, θ_min_ = 2.2°
Absorption correction: multi-scan (*SADABS*; Bruker, 2001)	*h* = −27→14
*T*_min_ = 0.712, *T*_max_ = 0.758	*k* = −8→7
7869 measured reflections	*l* = −24→23

### Refinement

**Table:**

Refinement on *F*^2^	Primary atom site location: structure-invariant direct methods
Least-squares matrix: full	Secondary atom site location: difference Fourier map
*R*[*F*^2^ > 2σ(*F*^2^)] = 0.021	Hydrogen site location: inferred from neighbouring sites
*wR*(*F*^2^) = 0.051	H atoms treated by a mixture of independent and constrained refinement
*S* = 1.03	*w* = 1/[σ^2^(*F*_o_^2^) + (0.0222*P*)^2^ + 3.2161*P*] where *P* = (*F*_o_^2^ + 2*F*_c_^2^)/3
2923 reflections	(Δ/σ)_max_ = 0.002
214 parameters	Δρ_max_ = 0.30 e Å^−^^3^
2 restraints	Δρ_min_ = −0.32 e Å^−^^3^

### Special details

**Table:**

Geometry. All e.s.d.'s (except the e.s.d. in the dihedral angle between two l.s. planes) are estimated using the full covariance matrix. The cell e.s.d.'s are taken into account individually in the estimation of e.s.d.'s in distances, angles and torsion angles; correlations between e.s.d.'s in cell parameters are only used when they are defined by crystal symmetry. An approximate (isotropic) treatment of cell e.s.d.'s is used for estimating e.s.d.'s involving l.s. planes.
Refinement. Refinement of *F*^2^ against ALL reflections. The weighted *R*-factor *wR* and goodness of fit *S* are based on *F*^2^, conventional *R*-factors *R* are based on *F*, with *F* set to zero for negative *F*^2^. The threshold expression of *F*^2^ > σ(*F*^2^) is used only for calculating *R*-factors(gt) *etc*. and is not relevant to the choice of reflections for refinement. *R*-factors based on *F*^2^ are statistically about twice as large as those based on *F*, and *R*- factors based on ALL data will be even larger.

### Fractional atomic coordinates and isotropic or equivalent isotropic displacement parameters (Å^2^)

**Table:**

	*x*	*y*	*z*	*U*_iso_*/*U*_eq_	
Ag1	0.301043 (8)	0.57692 (2)	0.020063 (9)	0.02934 (7)	
C1	0.37168 (10)	0.7372 (3)	0.16827 (11)	0.0247 (5)	
C2	0.43266 (10)	0.8225 (3)	0.21718 (11)	0.0232 (4)	
H2	0.4254	0.9535	0.2282	0.028*	
C3	0.47747 (10)	0.8155 (3)	0.17171 (11)	0.0241 (5)	
H3	0.4875	0.9436	0.1601	0.029*	
C4	0.43841 (10)	0.7149 (3)	0.10388 (11)	0.0236 (4)	
C5	0.46134 (9)	0.7102 (3)	0.28753 (11)	0.0229 (4)	
H5	0.4321	0.7026	0.3157	0.027*	
C6	0.47997 (9)	0.5181 (3)	0.26895 (11)	0.0224 (4)	
H6	0.4646	0.4077	0.2829	0.027*	
C7	0.17622 (12)	0.5344 (3)	0.07607 (13)	0.0352 (6)	
H11	0.2008	0.6046	0.1149	0.042*	
C8	0.11778 (13)	0.4724 (4)	0.07644 (15)	0.0419 (6)	
H12	0.1026	0.5036	0.1140	0.050*	
C9	0.08256 (12)	0.3642 (4)	0.02055 (15)	0.0437 (7)	
H13	0.0432	0.3190	0.0200	0.052*	
C10	0.10585 (11)	0.3222 (3)	−0.03534 (14)	0.0346 (5)	
H14	0.0826	0.2478	−0.0735	0.041*	
C11	0.16472 (10)	0.3932 (3)	−0.03348 (12)	0.0248 (5)	
C12	0.19220 (10)	0.3621 (3)	−0.09278 (12)	0.0254 (5)	
C13	0.16013 (11)	0.2632 (3)	−0.15462 (12)	0.0308 (5)	
H17	0.1206	0.2113	−0.1596	0.037*	
C14	0.18683 (12)	0.2423 (4)	−0.20821 (13)	0.0375 (6)	
H18	0.1655	0.1769	−0.2499	0.045*	
C15	0.24567 (12)	0.3190 (4)	−0.19984 (13)	0.0376 (6)	
H19	0.2647	0.3071	−0.2355	0.045*	
C16	0.27537 (11)	0.4139 (3)	−0.13678 (13)	0.0328 (5)	
H20	0.3151	0.4652	−0.1307	0.039*	
N1	0.19911 (9)	0.4976 (3)	0.02200 (10)	0.0282 (4)	
N2	0.25001 (8)	0.4358 (2)	−0.08404 (10)	0.0263 (4)	
N3	0.37858 (8)	0.6828 (2)	0.10383 (9)	0.0239 (4)	
O1	0.32354 (7)	0.7175 (2)	0.18440 (8)	0.0329 (4)	
O2	0.45990 (7)	0.6671 (3)	0.05599 (8)	0.0349 (4)	
O1W	0.43224 (10)	0.5971 (3)	−0.09313 (10)	0.0525 (5)	
H1A	0.4321 (17)	0.635 (5)	−0.0542 (13)	0.079*	
H1B	0.4609 (14)	0.519 (4)	−0.0847 (19)	0.079*	

### Atomic displacement parameters (Å^2^)

**Table:**

	*U*^11^	*U*^22^	*U*^33^	*U*^12^	*U*^13^	*U*^23^
Ag1	0.02316 (10)	0.03214 (11)	0.02658 (10)	−0.00518 (7)	−0.00091 (7)	0.00260 (7)
C1	0.0213 (11)	0.0274 (11)	0.0226 (11)	0.0055 (9)	0.0029 (9)	0.0072 (9)
C2	0.0231 (10)	0.0225 (11)	0.0217 (11)	0.0036 (9)	0.0036 (9)	0.0005 (9)
C3	0.0217 (10)	0.0265 (11)	0.0217 (11)	−0.0047 (9)	0.0035 (9)	0.0031 (9)
C4	0.0239 (11)	0.0263 (11)	0.0187 (10)	−0.0007 (9)	0.0039 (9)	0.0054 (9)
C5	0.0185 (10)	0.0311 (11)	0.0185 (10)	0.0023 (9)	0.0051 (8)	0.0007 (9)
C6	0.0194 (10)	0.0236 (10)	0.0197 (11)	−0.0024 (9)	−0.0002 (8)	0.0025 (9)
C7	0.0360 (13)	0.0386 (14)	0.0319 (13)	0.0003 (11)	0.0119 (11)	0.0047 (11)
C8	0.0434 (15)	0.0446 (15)	0.0464 (16)	0.0039 (12)	0.0265 (13)	0.0081 (13)
C9	0.0305 (13)	0.0442 (15)	0.0609 (18)	−0.0012 (12)	0.0209 (13)	0.0115 (14)
C10	0.0236 (11)	0.0329 (13)	0.0448 (15)	−0.0022 (10)	0.0075 (11)	0.0057 (11)
C11	0.0213 (11)	0.0225 (11)	0.0272 (12)	0.0007 (9)	0.0029 (9)	0.0070 (9)
C12	0.0217 (10)	0.0220 (10)	0.0286 (12)	0.0022 (9)	0.0024 (9)	0.0068 (9)
C13	0.0263 (12)	0.0278 (12)	0.0333 (13)	−0.0018 (10)	0.0021 (10)	0.0034 (10)
C14	0.0417 (14)	0.0345 (13)	0.0311 (13)	0.0000 (11)	0.0042 (11)	−0.0043 (11)
C15	0.0434 (14)	0.0402 (14)	0.0297 (13)	0.0053 (12)	0.0124 (11)	0.0003 (11)
C16	0.0276 (12)	0.0361 (13)	0.0349 (13)	−0.0011 (10)	0.0102 (10)	0.0036 (11)
N1	0.0256 (10)	0.0296 (10)	0.0285 (11)	−0.0012 (8)	0.0071 (8)	0.0045 (8)
N2	0.0215 (9)	0.0280 (10)	0.0264 (10)	−0.0010 (8)	0.0034 (8)	0.0027 (8)
N3	0.0208 (9)	0.0278 (10)	0.0202 (9)	−0.0011 (8)	0.0024 (7)	0.0031 (7)
O1	0.0215 (8)	0.0475 (10)	0.0298 (8)	0.0024 (7)	0.0081 (7)	0.0030 (8)
O2	0.0268 (8)	0.0563 (11)	0.0216 (8)	−0.0040 (8)	0.0076 (7)	−0.0017 (8)
O1W	0.0568 (13)	0.0680 (14)	0.0300 (10)	0.0172 (11)	0.0099 (10)	−0.0080 (10)

### Geometric parameters (Å, º)

**Table:**

Ag1—N3	2.1123 (17)	C7—H11	0.9300
Ag1—N2	2.2380 (18)	C8—C9	1.367 (4)
Ag1—N1	2.3510 (18)	C8—H12	0.9300
Ag1—Ag1^i^	3.2716 (4)	C9—C10	1.386 (4)
C1—O1	1.218 (2)	C9—H13	0.9300
C1—N3	1.377 (3)	C10—C11	1.394 (3)
C1—C2	1.520 (3)	C10—H14	0.9300
C2—C3	1.535 (3)	C11—N1	1.341 (3)
C2—C5	1.548 (3)	C11—C12	1.493 (3)
C2—H2	0.9800	C12—N2	1.350 (3)
C3—C4	1.520 (3)	C12—C13	1.390 (3)
C3—C5^ii^	1.539 (3)	C13—C14	1.370 (3)
C3—H3	0.9800	C13—H17	0.9300
C4—O2	1.230 (3)	C14—C15	1.380 (4)
C4—N3	1.352 (3)	C14—H18	0.9300
C5—C6	1.504 (3)	C15—C16	1.381 (3)
C5—C3^ii^	1.539 (3)	C15—H19	0.9300
C5—H5	0.9800	C16—N2	1.336 (3)
C6—C6^ii^	1.330 (4)	C16—H20	0.9300
C6—H6	0.9300	O1W—H1A	0.81 (3)
C7—N1	1.340 (3)	O1W—H1B	0.82 (3)
C7—C8	1.376 (3)		
			
N3—Ag1—N2	157.77 (7)	C9—C8—H12	120.6
N3—Ag1—N1	129.00 (7)	C7—C8—H12	120.6
N2—Ag1—N1	72.09 (7)	C8—C9—C10	119.6 (2)
N3—Ag1—Ag1^i^	104.80 (5)	C8—C9—H13	120.2
N2—Ag1—Ag1^i^	90.12 (5)	C10—C9—H13	120.2
N1—Ag1—Ag1^i^	65.33 (5)	C9—C10—C11	119.0 (2)
O1—C1—N3	124.7 (2)	C9—C10—H14	120.5
O1—C1—C2	124.46 (19)	C11—C10—H14	120.5
N3—C1—C2	110.86 (17)	N1—C11—C10	120.9 (2)
C1—C2—C3	103.58 (16)	N1—C11—C12	116.48 (18)
C1—C2—C5	113.16 (18)	C10—C11—C12	122.6 (2)
C3—C2—C5	109.94 (17)	N2—C12—C13	120.9 (2)
C1—C2—H2	110.0	N2—C12—C11	116.92 (19)
C3—C2—H2	110.0	C13—C12—C11	122.1 (2)
C5—C2—H2	110.0	C14—C13—C12	119.9 (2)
C4—C3—C2	103.13 (16)	C14—C13—H17	120.1
C4—C3—C5^ii^	113.28 (18)	C12—C13—H17	120.1
C2—C3—C5^ii^	110.16 (16)	C13—C14—C15	119.4 (2)
C4—C3—H3	110.0	C13—C14—H18	120.3
C2—C3—H3	110.0	C15—C14—H18	120.3
C5^ii^—C3—H3	110.0	C14—C15—C16	117.9 (2)
O2—C4—N3	125.1 (2)	C14—C15—H19	121.1
O2—C4—C3	123.12 (19)	C16—C15—H19	121.1
N3—C4—C3	111.81 (18)	N2—C16—C15	123.5 (2)
C6—C5—C3^ii^	107.48 (16)	N2—C16—H20	118.3
C6—C5—C2	108.60 (16)	C15—C16—H20	118.3
C3^ii^—C5—C2	105.19 (17)	C7—N1—C11	119.3 (2)
C6—C5—H5	111.8	C7—N1—Ag1	125.20 (16)
C3^ii^—C5—H5	111.8	C11—N1—Ag1	115.26 (14)
C2—C5—H5	111.8	C16—N2—C12	118.4 (2)
C6^ii^—C6—C5	114.88 (11)	C16—N2—Ag1	122.89 (15)
C6^ii^—C6—H6	122.6	C12—N2—Ag1	118.63 (15)
C5—C6—H6	122.6	C4—N3—C1	110.35 (17)
N1—C7—C8	122.6 (2)	C4—N3—Ag1	128.63 (14)
N1—C7—H11	118.7	C1—N3—Ag1	120.98 (14)
C8—C7—H11	118.7	H1A—O1W—H1B	105 (4)
C9—C8—C7	118.7 (2)		
			
O1—C1—C2—C3	−179.7 (2)	C12—C11—N1—C7	178.35 (19)
N3—C1—C2—C3	1.2 (2)	C10—C11—N1—Ag1	174.03 (16)
O1—C1—C2—C5	61.3 (3)	C12—C11—N1—Ag1	−7.3 (2)
N3—C1—C2—C5	−117.83 (19)	N3—Ag1—N1—C7	9.0 (2)
C1—C2—C3—C4	−3.7 (2)	N2—Ag1—N1—C7	−179.1 (2)
C5—C2—C3—C4	117.52 (18)	Ag1^i^—Ag1—N1—C7	−80.37 (18)
C1—C2—C3—C5^ii^	−124.88 (18)	N3—Ag1—N1—C11	−164.88 (14)
C5—C2—C3—C5^ii^	−3.7 (2)	N2—Ag1—N1—C11	7.03 (15)
C2—C3—C4—O2	−173.5 (2)	Ag1^i^—Ag1—N1—C11	105.71 (16)
C5^ii^—C3—C4—O2	−54.5 (3)	C15—C16—N2—C12	−0.2 (3)
C2—C3—C4—N3	5.4 (2)	C15—C16—N2—Ag1	176.70 (18)
C5^ii^—C3—C4—N3	124.47 (19)	C13—C12—N2—C16	1.0 (3)
C1—C2—C5—C6	64.1 (2)	C11—C12—N2—C16	−178.63 (19)
C3—C2—C5—C6	−51.2 (2)	C13—C12—N2—Ag1	−176.10 (16)
C1—C2—C5—C3^ii^	178.93 (17)	C11—C12—N2—Ag1	4.3 (2)
C3—C2—C5—C3^ii^	63.66 (18)	N3—Ag1—N2—C16	−19.6 (3)
C3^ii^—C5—C6—C6^ii^	−56.6 (3)	N1—Ag1—N2—C16	177.18 (19)
C2—C5—C6—C6^ii^	56.7 (3)	Ag1^i^—Ag1—N2—C16	113.25 (17)
N1—C7—C8—C9	2.1 (4)	N3—Ag1—N2—C12	157.30 (17)
C7—C8—C9—C10	−1.0 (4)	N1—Ag1—N2—C12	−5.89 (15)
C8—C9—C10—C11	−0.6 (4)	Ag1^i^—Ag1—N2—C12	−69.82 (15)
C9—C10—C11—N1	1.2 (3)	O2—C4—N3—C1	174.0 (2)
C9—C10—C11—C12	−177.3 (2)	C3—C4—N3—C1	−4.9 (2)
N1—C11—C12—N2	2.3 (3)	O2—C4—N3—Ag1	−8.5 (3)
C10—C11—C12—N2	−179.1 (2)	C3—C4—N3—Ag1	172.56 (13)
N1—C11—C12—C13	−177.2 (2)	O1—C1—N3—C4	−176.8 (2)
C10—C11—C12—C13	1.4 (3)	C2—C1—N3—C4	2.3 (2)
N2—C12—C13—C14	−1.1 (3)	O1—C1—N3—Ag1	5.4 (3)
C11—C12—C13—C14	178.5 (2)	C2—C1—N3—Ag1	−175.45 (13)
C12—C13—C14—C15	0.4 (4)	N2—Ag1—N3—C4	15.3 (3)
C13—C14—C15—C16	0.3 (4)	N1—Ag1—N3—C4	174.54 (16)
C14—C15—C16—N2	−0.4 (4)	Ag1^i^—Ag1—N3—C4	−115.44 (17)
C8—C7—N1—C11	−1.4 (4)	N2—Ag1—N3—C1	−167.45 (16)
C8—C7—N1—Ag1	−175.12 (18)	N1—Ag1—N3—C1	−8.19 (19)
C10—C11—N1—C7	−0.3 (3)	Ag1^i^—Ag1—N3—C1	61.83 (16)

Symmetry codes: (i) −*x*+1/2, −*y*+3/2, −*z*; (ii) −*x*+1, *y*, −*z*+1/2.

### Hydrogen-bond geometry (Å, º)

**Table:**

*D*—H···*A*	*D*—H	H···*A*	*D*···*A*	*D*—H···*A*
O1*W*—H1*A*···O2	0.81 (3)	2.07 (2)	2.839 (2)	159 (3)
O1*W*—H1*B*···O2^iii^	0.82 (3)	2.13 (3)	2.952 (3)	175 (2)

Symmetry code: (iii) −*x*+1, −*y*+1, −*z*.
